# Differences in Measured and Self-Categorized Food Security Status and Related Coping Strategies among College Students

**DOI:** 10.3390/nu14173569

**Published:** 2022-08-30

**Authors:** Megan D. Engel, Karla P. Shelnutt, Lisa A. House, Aseel El Zein, Anne E. Mathews

**Affiliations:** 1 Food Science and Human Nutrition Department, University of Florida, Gainesville, FL 32611, USA; 2 Department of Family, Youth, and Community Sciences, University of Florida, Gainesville, FL 32611, USA; 3 Department of Food and Resource Economics Department, University of Florida, Gainesville, FL 32611, USA; 4 Department of Nutrition Sciences, University of Alabama at Birmingham, Birmingham, AL 35233, USA

**Keywords:** food insecurity, nutrition insecurity, coping strategies, college students, obesity, young adults, USDA AFSSM

## Abstract

Qualitative studies suggest that college students with food insecurity (FI) experience stigma and misinterpret some of the USDA Adult Food Security Survey Module (AFSSM) questions, leading to misclassification of food security (FS) status. We aimed to evaluate differences in AFSSM-measured FS status and self-categorized FS status (based on USDA descriptions of the four FS levels) among college students, and to identify differences in the coping strategies and BMI of these students. Data were collected cross-sectionally from a convenience sample via web-based, self-reported surveys. Measured FS, self-categorized FS, coping strategies, and self-reported BMI were key variables of interest. Participants were 1003 undergraduate and graduate students (22.2 ± 4.6 years; 65.7% female). Of the participants measured as food insecure (40.0%), 57.8% self-categorized as food secure (MFI-SFS) and 42.2% self-categorized as food insecure (MFI-SFI). Significantly more MFI-SFI participants were AFSSM-categorized as having very low FS when compared to MFI-SFS participants (71.6% vs. 46.6%, *p* < 0.05). MFI-SFI participants reported significantly higher BMI (M = 24.7, SD ± 6.0 kg/m^2^) and coping strategies scores (M = 49.8, SD ± 7.5) when compared to MFI-SFS participants (M = 23.1, SD ± 3.6 kg/m^2^; M = 46.9, SD ± 7.5, respectively, *p* ≤ 0.01). Assessment of and interventions to address FI among college students should consider the potential influence of self-perception and students’ interpretation of survey questions.

## 1. Introduction

Food insecurity (FI), defined as the inability to consistently access enough nutritionally adequate and safe food through socially acceptable means, is commonly assessed in a variety of populations, including college students, using the United States Department of Agriculture (USDA) Adult Food Security Survey Module (AFSSM) [1,2]. Two systematic reviews of post-secondary student FI conducted before COVID-19 reported average rates of 35% and 43%, which are significantly higher than the 10.5% of households in the United States (US) with FI in 2019 [3,4,5]. Other peer-reviewed studies report pre-COVID-19 FI rates among US college students ranging from 14 to 59% [4,6,7]. This wide range of FI prevalence may be influenced in part by the variations in assessment methods among studies. Some studies have utilized select questions from the validated AFSSM with an altered time frame referenced in the questions [8]. However, most studies have used various versions of the USDA Food Security Survey Module (6-item AFSSM, 10-item AFSSM, or 18-item household food security module). The use of these semi-structured and more objective measures of food security (FS) can be useful when quantification of FS is necessary. However, these tools can only capture part of the picture. The incorporation of behavioral assessment and qualitative measures of FS are equally important in understanding FI and identifying the less tangible elements that influence it.

Previous research in other special populations has suggested that individuals’ experiences likely influence their perception of FS and impact how they respond to FS questions [9,10,11]. This research also suggests that the AFSSM should be adapted for special populations due to the unique influencers of food access and FS these populations experience which lead to the potential under or over-estimation of FI with the current AFSSM tool [10,11]. College students have similarly unique influencers of food access. This could explain, in part, why inconsistencies in response patterns of college students have been found when evaluating results of different versions of the AFSSM [12]. These inconsistencies could also be due to misalignment of students’ perception of FS and their AFSSM-measured FS status, similar to the misalignment seen in other special populations. Additionally, the AFSSM and other tools developed to measure FS have not been validated in college students [12]. These FS tools are also unable to calculate and consider the inherent complexity of the college student population (large ranges in food and financial support, employment status, and resources; inconsistent income and housing in a 12-month period; etc.). These less tangible factors that are not captured in the widely utilized FS measurement tools make the seemingly straightforward questions difficult to answer for college students. As a result, students may be providing convenient, albeit less accurate, responses [12]. Nikolaus et al. ultimately found that the 10-item AFSSM with the 2-item screener was the most accurate method of assessing FS status in college students, but even this version was less than ideal [12,13].

Regardless of discrepancies in prevalence, college students AFSSM-measured as food insecure report behaviors that adversely affect their health. Compared to their food secure peers, students experiencing FI report lower food preparation skills and cooking self-efficacy [14], poorer diet quality [15], increased risk of obesity [15], decreased sleep quality [16], more disordered eating behaviors [16], and poorer psychosocial health status and academic performance [15,16,17]. College students with AFSSM-measured FI also tend to utilize more coping strategies to acquire food and to maintain an adequate food supply [18]. These coping strategies can involve saving money (e.g., using less utilities), using support systems (e.g., using a food bank), altering quality or intake of food (e.g., purchasing cheap, processed food), and selling items for food money (e.g., selling textbooks) [18,19]. Utilization of these coping strategies, along with the common misperceptions that consistent access to nutritious foods is a luxury while in college, may falsely inflate an individual’s perception of FS while in college, leading to students’ hesitation to seek assistance [19]. Multiple studies suggest that many college students with FI do not utilize available support services, such as on-campus food pantries, due to psychosocial barriers including social stigma, feeling that others need the resources more, as well as the normalization of limited access to nutritious food during college [16,20,21]. When asked about their financial circumstances, students with FI report that food-related expenses are a discretionary component of their budget due to the rising cost of living, limited income, financial independence, and reliance on financial aid and student loans [22]. Undoubtedly, rising college tuition costs and other essential expenses such as textbooks and housing leave these students with a limited budget to purchase nutritious food [22]. All of these intangible factors add to the complexity of identifying FS in college students. Further research into college students’ perceptions of FS is warranted with close examination of students’ food-related coping strategies to determine if these factors influence how students perceive their FS status. The purpose of this study was to (1) evaluate differences between students’ AFSSM-measured and self-categorized FS status and (2) identify any differences in pertinent coping strategies and BMI of students with AFSSM-measured FI who categorize themselves as food secure and food insecure.

## 2. Materials and Methods

### 2.1. Study Design and Participants

Using convenience sampling, a cross-sectional, web-based survey was distributed via email to all students enrolled in on-campus classes at the University of Florida in the United States during the Summer 2019 semester. An invitation to complete the survey along with the survey link were emailed three times and the survey was open from 16 May to 30 May 2019. The survey was delivered via Qualtrics, a secured online survey platform (2022, Qualtrics LLC). Undergraduate and graduate students 18 years of age and older were eligible to participate. Before beginning the survey, each student was asked to provide informed consent electronically. Every third student who completed the survey received USD 10 redeemable at on-campus dining venues. This study was deemed exempt by the Institutional Review Board of the University of Florida. The study was conducted in accordance with the Declaration of Helsinki and approved by the Institutional Review Board of the University of Florida (IRB #201901188, approved 29 April 2019).

Participants were informed of the purpose of the study, the study’s ethical approval information, and their rights as a study participant. If the student agreed to participate, they were sent to the beginning of the survey questions. The survey assessed AFSSM-measured and self-categorized FS status, financial coping strategies, food purchasing and acquisition behaviors, self-reported height, weight, and sociodemographic characteristics. Two validation questions were included in the survey to address potential misreporting by participants. The first validation question, “Please answer “somewhat agree” for this row. Thank you for reading carefully.” was placed within a block of questions asking about behaviors related to food pantries that appears shortly after the first section of the survey. The second validation question, “Please answer “sometimes” for this question. Thank you for reading carefully.” was placed within the coping strategies questions. Upon completion, participants were directed to a webpage with available campus and local food assistance resources available to them. 

### 2.2. Measures

#### 2.2.1. Participant Characteristics

Participant characteristics including age, height, and weight (used to calculate BMI), gender identity, race/ethnicity, marital status, year in college, employment status, receipt of a Pell grant, place of residence (on-campus/off-campus), academic residence status (in-state, out-of-state, international), and financial independence were collected.

#### 2.2.2. Food Security Status

AFSSM-measured Food Security Status: FS status was measured using the 10-item USDA AFSSM (α = 0.743; κ = 0.69) [1,23]. Each item asks participants about their food situation in the past 12 months with questions covering topics including having enough money for food, hunger due to not having money for food, and other FS status indicators. The survey yields a total raw score ranging from 0 to 10, with one point awarded to each item with an affirmative response. Respondents are categorized as having high FS (raw score of 0), marginal FS (raw score of 1–2), low FS (raw score of 3–5), and very low FS (raw score of 6–10) based on their responses. The USDA defines individuals with high FS as those who report no food access related problems or limitations. Individuals with marginal FS may have some anxiety or worry about food running out, but the quality and amount of food consumed is unaffected. Low FS refers to individuals whose quality, variety, or desirability of diet decreases but quantity of food is unaffected. Individuals with very low FS report multiple indications of disrupted eating patterns and decreased food intake [2]. These four categories describe a range of potential FS statuses; however, individuals are often recategorized into two groups: food secure (combining high FS and marginal FS) and food insecure (low FS and very low FS) [2].

Self-categorized Food Security Status: To capture the participants’ perceptions of their FS status, they were provided the USDA AFSSM category definitions for high, marginal, low, and very low FS (described above) in addition to the statement “The US Department of Agriculture describes a person’s food security status using the four categories shown below”. Participants were asked “Which category do you think you belong in?”. Reponses to this question were treated as a participant’s self-categorized FS status. After categorizing themselves based on the USDA FS category definitions, participants were informed of their AFSSM-measured FS status within the survey process. 

#### 2.2.3. Coping Strategies

Participants’ coping strategies were assessed by asking “what strategies have you used to ensure that you have adequate and sufficient access to food?” with 28 potential strategies listed and response options of “often,” “sometimes,” or “never” for each strategy. This coping strategies scale (CSS) was previously adapted from literature examining coping strategies of populations with FI, including college students [18,19]. Listed strategies were focused within four categories: saving, support, food access, and selling. Responses to the 28-item CSS were scored by allotting one point for a response of “never,” two points for “sometimes,” and three points for “often.” Total coping strategy scores can range from 28 to 84 points, where a higher score means participants are employing more coping strategies. To limit inaccurate responses within this survey block, a survey validation question was added where participants were asked to “please answer ‘sometimes’ for this question” [24]. Survey data of participants who failed this test question were removed before analysis.

### 2.3. Statistical Analysis

Sociodemographic characteristics of participants were compared by AFSSM-measured FS status using χ^2^ tests for categorical variables and independent samples *t*-tests for continuous variables. Participants’ AFSSM-measured FS status were determined using the USDA AFSSM scoring where all affirmative responses (yes; often; sometimes; almost every month; some months but not every month) were coded as one point and a total score was summated [1]. Next, the USDA AFSSM raw score ranges for low (3–5) and very low (6–10) FS were used to create a dichotomous categorical variable. This variable was then used to analyze prevalence of participants AFSSM-measured as food insecure who categorized themselves as food secure (MFI-SFS) and AFSSM-measured as food insecure who categorized themselves as food insecure (MFI-SFI) using χ^2^ tests. indicates that Differences in mean overall CSS scores, subscale scores, and BMI between FS categories were analyzed using independent samples *t*-tests. To further understand differences in overall CSS scores among MFI-SFS and MFI-SFI participants, CSS scores were separated into quartiles and compared using χ^2^ tests. All participants who successfully completed the survey were included in analysis. Missing data for each outcome were reported in the tables and figures. A *p*-value of <0.05 was considered statistically significant, and all statistical tests were two-sided. Data were analyzed using SPSS Statistics for Windows, version 26.0.0.0 (2019, IBM-SPSS Statistics Inc, Armonk, NY, USA).

## 3. Results

### 3.1. Participant Characteristics

During the Summer 2019 semester, 28,193 undergraduate and graduate students were enrolled in on-campus courses and were potentially eligible to participate. Duplicate responses identified via students’ university email addresses were removed and data were de-identified prior to analysis. A total of 1406 students started the survey, and 1003 (71.3%) students successfully completed the survey and were included in analysis. Of the non-completers (n = 403), 9 exited the survey without answering any questions, 8 exited while reviewing the IRB waiver, 4 students were under the age of 18, 76 participants failed the survey validation questions, and the remaining 306 did not finish the survey completely. Participants (n = 1003) were predominantly female (65.7%), non-Hispanic/Latino (77.4%), and white (67.8%). The average age of the sample was 22.2 ± 4.6 years, most were undergraduate students (86.4%), and the majority reported living in off-campus housing (84.2%). Approximately one quarter of participants were Pell grant recipients (28.5%), and 41.4% of participants reported being financially independent. A significantly higher proportion of participants with FI were Hispanic/Latino (28.7%) and Black/African American (12.8%) when compared to the proportion of participants AFSSM-measured as food secure (18.5% Hispanic/Latino, 3.5% Black/African American). Of the participants AFSSM-measured as food insecure, a higher proportion reported being Pell grant recipients and financially independent when compared to those measured as food secure (Table 1).

### 3.2. AFSSM-Measured Food Security Compared with Self-Categorized Food Security Status

Using the AFSSM, 60.0% of participants were categorized as food secure (37.9% high FS; 22.1% marginal FS). The remaining 40.0% were categorized as food insecure (17.2% low FS; 22.8% very low FS). When asked about what USDA category they fall in, 80.2% of participants chose the ‘food secure’ categories (53.0% self-categorized high FS; 27.2% self-categorized marginal FS) while only 19.8% categorized themselves as food insecure (16.1% self-categorized low FS; 3.7% self-categorized very low FS). Of those who were AFSSM-measured as highly food secure, over 90% categorized themselves as highly food secure. The largest proportion of the participants were AFSSM-measured as food secure (either high or marginal FS status) and self-categorized as food secure (MFS-SFS; n = 572; 57.0%).

**Figure 1:**
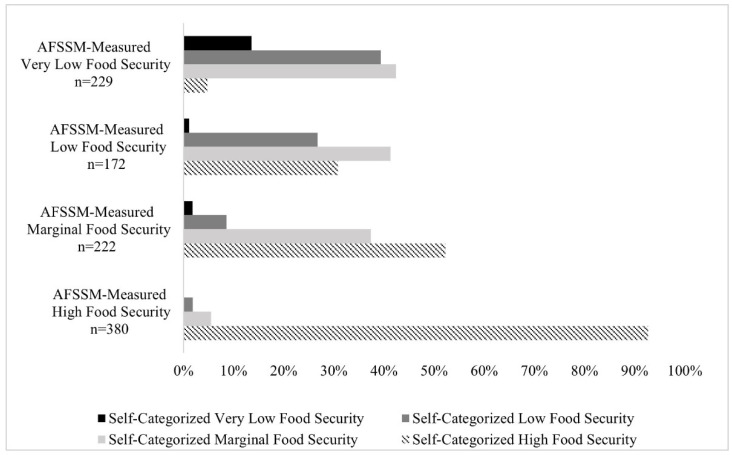
Self-Categorized Food Security Status and USDA AFSSM-Measured Food Security Status among College Students (n = 1003). USDA AFSSM = US Department of Agriculture 10-item Adult Food Security Survey Module.

Of those who had very low AFSSM-measured FS, less than 15% perceived themselves as having very low FS with about 40% categorizing themselves with low FS and another 42% categorizing themselves as marginally food secure (Figure 1). Participants AFSSM-measured as food secure who categorized themselves as food insecure made up the smallest group (MFS-SFI; n = 30; 3.0%). The remaining two groups—participants AFSSM-measured as food insecure who categorized themselves as food secure (MFI-SFS; n = 232; 23.1%), and participants AFSSM-measured as food insecure who categorized themselves as food insecure (MFI-SFI; n = 169; 16.9%)—were the focus of the remaining analyses to better understand differences in self-categorization among participants AFSSM-measured as food insecure.

### 3.3. Differences in AFSSM Question Responses between Food Insecure Students Who Self-Categorized as Food Secure and Food Insecure

**Figure 2:**
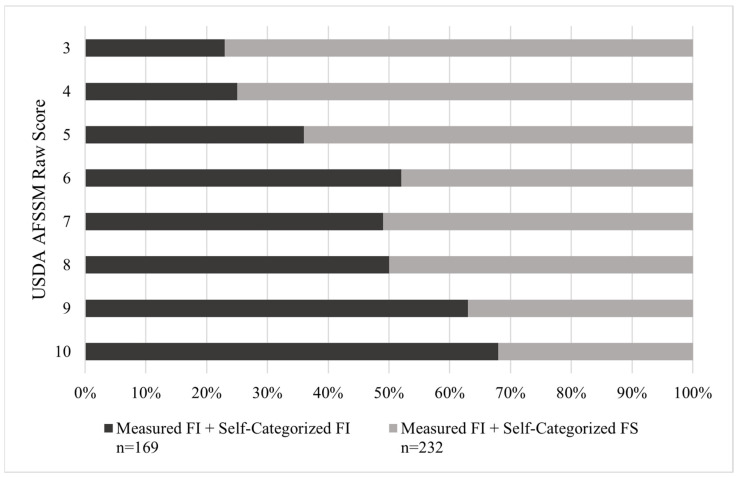
Raw Scores of USDA AFSSM by Self-Categorized Food Security Status among College Students AFSSM-Measured as Food Insecure (n = 401). USDA AFSSM = US Department of Agriculture 10-item Adult Food Security Survey Module; FI = Food Insecure; FS = Food Secure.

To better understand this discrepancy between the participants who were AFSSM-measured as food insecure who self-categorized as food secure (MFI-SFS) and those who were AFSSM-measured as food insecure who categorized themselves as food insecure (MFI-SFI), differences in their responses to the 10-item AFSSM questions were analyzed. There were significant differences in AFSSM question responses between MFI-SFS and MFI-SFI participants for all AFSSM questions except for two: AD4 (“In the last 12 months, did you lose weight because there wasn’t enough money for food?”) and AD5a (If affirmative response to AD5, “How often did this [not eating for a whole day because there wasn’t enough money for food] happen?”). A higher proportion of MFI-SFI participants answered each question (besides AD4 and AD5a) with an affirmative response (Supplemental Table S1). Similarly, when looking at the range of possible raw AFSSM scores for individuals measured as food insecure, a significantly higher proportion of MFI-SFI individuals had raw scores towards the upper end of the scoring range (score of 6–10), compared to MFI-SFS individuals. Inversely, a significantly higher proportion of MFI-SFS individuals scored in the lower end of the range (score of 3–5) (Figure 2; Table 2; χ^2^ (1, n = 401) = 25.0, *p* < 0.001).

### 3.4. Differences in Coping Strategies, and BMI

Participants AFSSM-measured as food insecure (MFI) in this study reported higher use of coping strategies overall (MFI M = 48.1, SD ± 7.6; MFS M = 39.3, SD ± 6.7; *p* < 0.001) and higher use of coping strategies related to saving (MFI M = 18.0, SD ± 3.7; MFS M = 14.3, SD ± 3.5; *p* < 0.001), support (MFI M = 16.3, SD ± 3.1; MFS M = 13.9, SD ± 2.6; *p* < 0.001), food intake/access (MFI M = 8.7, SD ± 1.7; MFS M = 6.9, SD ± 1.6; *p* < 0.001), and selling (MFI M = 5.1, SD ± 1.5; MFS M = 4.3, SD ± 0.8; *p* < 0.001), when compared to participants AFSSM-measured as food secure (MFS). Coping strategies scores for AFSSM-measured food insecure participants differed by their self-categorization. When compared to those who categorized themselves as food secure (MFI-SFS), those who self-categorized as food insecure (MFI-SFI) reported significantly higher overall CSS scores (MFI-SFI M = 49.8, SD ± 7.5; MFI-SFS M = 46.9, SD ± 7.5; *p* < 0.001) as well as higher scores for three of the four subscales: saving (MFI-SFI M = 18.8, SD ± 3.5; MFI-SFS M = 17.4, SD ± 3.7; *p* < 0.001), access (MFI-SFI M = 9.1, SD ± 1.6; MFI-SFS M = 8.4, SD ± 1.7; *p* < 0.001), and selling (MFI-SFI M = 5.4, SD ± 1.6; MFI-SFS M = 5.0, SD ± 1.4; *p* = 0.01) (Table 3). When separating the overall CSS score into four quartiles, the highest proportion of the MFI-SFS group (n = 70; 31.4%) scored in the lowest quartile suggesting these MFI-SFS participants utilize a low number of coping strategies while the MFI-SFI group had the highest proportion of individuals (n = 49; 29.7%) scoring in the highest quartile (Table 4; χ^2^ (3, n = 388) = 14.8, *p* = 0.002). 

To further understand these differences among participants AFSSM-measured as food insecure, measured FI was further separated into low and very low measured FS and overall CSS score was compared among the four different groups: (1) measured low FS, self-categorized as food secure (low FS-SFS); (2) measured low FS, self-categorized as food insecure (low FS-SFI); (3) measured very low FS, self-categorized as food secure (very low FS-SFS); (4) measured very low FS, self-categorized as food insecure (very low FS-SFI). The significant differences in overall CSS scores are seen mostly between the low FS-SFS and very low FS-SFI groups. A significantly higher proportion of very low FS-SFI participants (n = 42; 35.3% of group) scored in the highest overall CSS score quartile when compared to the low FS-SFS group (n = 11; 9.3% of group; *p* < 0.05). As expected, a significantly higher proportion of low FS-SFS (n = 43; 36.8% of low FS-SFS group) had an overall CSS score in the lowest quartile when compared to the very low FS-SFI group (n = 19; 16.0% of very low FS-SFI group; *p* < 0.05). The very low FS-SFI group had a significantly higher proportion of individuals in the highest quartile when compared to all groups, suggesting that this group utilizes the most coping strategies (Supplemental Table S2; χ^2^ (9, n = 388) = 38.9, *p* < 0.001).

There were also significant differences in some individual CSS questions between MFI-SFS and MFI-SFI groups. A significantly higher proportion of MFI-SFI individuals reported taking fewer classes, using less utilities (electricity, water), saving money on medicines or medical appointments, stretching food to last longer, saving food for emergencies, obtaining food from food banks or pantries, eating less healthy meals to eat more food, purchasing cheap and processed food, and selling personal possessions as strategies to ensure they have adequate and sufficient access to food (Supplemental Table S3).

Students who categorized themselves as food insecure and were AFSSM-measured as food insecure (MFI-SFI) reported significantly higher BMI (24.7 ± 6.0 kg/m^2^) than those who categorized themselves as food secure and were AFSSM-measured as food insecure (MFI-SFS; 23.1 ± 5.6 kg/m^2^; *p* = 0.002). When examined more closely, this difference stems from a significant difference in BMI among women who measured and self-categorized as FI (MFI-SFI) compared with women who measured as FI, but self-categorized as FS (MFI-SFS) (24.9 ± 6.4 kg/m^2^; 22.7 ± 22.7 kg/m^2^; *p* = 0.002).

## 4. Discussion

Findings in this study suggest significant discrepancies between USDA AFSSM categorization of FS status and self-categorized FS status among college students and highlights the need for a validated FS scale for this population. The largest discrepancies are seen in those participants who were AFSSM-measured as food insecure. Less than half (42.1%) of participants AFSSM-measured as food insecure also categorized themselves as food insecure (MFI-SFI). Of those who self-identified as food insecure (MFI-SFI), a higher proportion categorized themselves with very low FS when compared to those that were measured as food insecure but self-categorized as food secure (MFI-SFS), suggesting that these MFI-SFI individuals could be experiencing FI more frequently and/or more severely. MFI-SFI participants also reported the highest mean BMI and reported utilizing significantly more coping strategies related to saving, selling, and food intake/access compared to MFI-SFS participants. This highlights the complicated relationship between stable access to healthful foods, food choices, and health disparities.

This study is the first to quantitatively measure differences in AFSSM-measured and self-categorized FS status among college students. While the study is unable to directly identify the reasons for the discrepancy between AFSSM-measured and self-categorized food security status, the literature points to two plausible factors: college students’ understanding of the questions included in the USDA AFSSM, and stigma associated with experiencing FI. Previous literature has questioned the accuracy of measuring FS among college students using the USDA AFSSM [12,25]. FS rates differ with different versions of FS surveys, with higher FI estimates among college students using the USDA AFSSM when compared to the USDA Household Food Security Survey Module, and differences in rates depending on the reference period (12 months or ≤9 months) [13]. Qualitative data indicate that college students do not interpret questions in the AFSSM in the same way as other adults, with difficulties interpreting the “money for more” clauses of each question and difficulties addressing questions about balanced meals and weight loss related to insufficient food [25]. The term “balanced meals” used in the AFSSM phrase “I couldn’t afford to eat balanced meals” has been identified as an unreliable phrase in other populations due the heterogeneity of the interpretation of “balanced” [26]. College students and affluent individuals may compare their diets to a higher standard of “balanced,” focusing on the Dietary Guidelines for Americans [27] with consideration of different food groups and high diet quality. However, the term “balanced” in the context of the AFSSM relates to adequate intake of essential minerals and vitamins, not optimal diet quality, with the goal of identifying individuals experiencing undernutrition and malnutrition [28]. In this study, regardless of how they categorized themselves, most students with AFSSM-measured FI reported that they sometimes or often cannot afford to eat balanced meals. However, more MFI-SFI participants reported that they often experience the inability to afford balanced meals when compared to MFI-SFS participants. In general, MFI-SFI participants reported more frequent signs of FI when compared to MFI-SFS participants, with higher raw scores on the 10-item AFSSM (indicating more affirmative responses), and more individuals reporting “often true” to questions asking about running out of food, being worried about food, and inability to afford balanced meals (HH2-HH4). Ellison et al. present an important viewpoint on FI among college students to consider. Because college students are such a heterogenous population, there are unique considerations which may not be captured by current FS measures. First, does the student live on campus and does the university require students to purchase a meal plan while on campus? Is the student financially independent or do they rely on family, financial aid, free food, and/or other resources? Does the student rely on food acquisition-related coping strategies to acquire food? Are there other factors besides financial that contribute to FI (e.g., time constraints)? Students often have inconsistent schedules and may experience changes in access to resources throughout the year [29]. Each of these factors may introduce unique barriers students experience that prevent them from accessing adequate, nutritious food but they are not directly addressed with current FS measures.

In this cohort, MFI-SFI participants appear to experience more severe FI when compared to MFI-SFS participants. Although potential misinterpretation of the AFSSM questions may be one potential explanation for these differences in self-categorization, another potential explanation for the differences in these two groups may be due to psychosocial barriers the MFI-SFS group experiences. Previous literature suggests some college students with FI do not seek support due to several psychosocial barriers. Some of these barriers include social stigma, perceptions that others need the resources more than them, and that their experience is part of the normal college experience [16,20,21]. El Zein et al. conducted in depth interviews with 41 college students regarding barriers to using an on-campus food pantry [30]. This study suggests that the primary reason students experiencing FI (as measured by the USDA AFSSM) were not using the pantry was feelings of stigma and shame. Stigma and shame along with other psychosocial barriers may lead to an inability to self-identify FI, especially if FI is not experienced as consistently or severely.

Student participants AFSSM-measured as food insecure in this study also reported higher use of food acquisition coping strategies related to all four subscales (saving, support, food intake/access, and selling) as well as overall coping strategies score, when compared to student participants measured as food secure. However, MFI-SFI participants reported the highest usage of coping strategies overall with higher usage of saving, food intake/access, and selling-related strategies when compared to MFI-SFS participants. Previous literature supports these results, reporting higher food acquisition coping strategies as predictors of FI in college students and adults with low income [18,19]. These differences in coping strategies between MFI-SFI and MFI-SFS participants may provide some insights into better understanding FI in college students. Incorporation of additional questions about food acquisition-related coping strategies and behaviors may aid in more accurate estimations of the prevalence of FI among college students. 

Individuals who are AFSSM-measured as food insecure and categorize themselves as food insecure (MFI-SFI) may be experiencing more frequent and severe FI, which may result in utilization of more coping strategies to maintain an adequate food supply. Collecting both AFSSM-measured and self-categorized FS status may help identify students that are currently receptive, and those less receptive, to utilizing food acquisition resources, such as a food pantry. Targeted messages to reduce stigma and bias could be developed to reduce these potential barriers, allowing students to access available resources more often. Identifying and tailoring FS-related interventions may be an effective way of alleviating FI in college students. Further research focused on developing and testing additional and alternative questions for assessing FI among college students is warranted, with consideration of questions involving students’ self-categorization and coping strategies.

The findings of the current study should be interpreted with consideration of limitations in the study design. First, this study was cross-sectional in design which prevents the ability to draw conclusions about causal relationships between FI and coping strategies among college students. The data from this study were collected from a convenience sample of college students at one large, public university in the United States which may limit the generalizability to all college students. The sample population may also have over-represented those who have an interest in food access issues and/or those who are in financial need (compensation was available for survey completers). Second, since FI can be perceived as socially undesirable, an important limitation is social desirability bias [31]. Participants might have responded to survey questions in a way they perceived as socially acceptable and favorable—especially when self-identifying their FS status and reporting use of coping strategies that are not considered socially acceptable. It is also important to note that, despite the historical use and evidence supporting use of the AFSSM in the general population, it has not been validated in the college setting [32]. Further studies are needed to assess the psychometric properties of the AFSSM among college students to ensure accurate estimates of the prevalence of FI among this population. Accurate assessment of FS status of college students is imperative as campus administrators and policymakers may use these data to allocate resources for students. Third, weight and height were self-reported which may have rendered the derived BMI results unreliable due to lack of recall. Lastly, data from this study were also collected prior to the COVID-19 pandemic. Recent literature has suggested that COVID-19 and its economic impact have increased FI rates among college students and may alter the key factors that are leading to FI in this population [33].

## 5. Conclusions

A significant portion of college students’ self-categorized FS status differed from their FS status measured by the USDAAFSSM. Findings from this study suggest that students who are categorized as food insecure by the AFSSM and categorized themselves as food insecure (MFI-SFI) are more likely to employ a higher number of coping strategies related to food acquisition. Although the results of this study may not be generalizable to other colleges and universities, this study provides a snapshot of the food security status and coping strategies of college students at a large, public, southeastern university. Overall, future studies should investigate and consider two potential factors influencing these differences in measured and self-categorized FS status seen among college students. First, college students’ interpretation of questions in the USDA AFSSM should be further examined and the potential need for a college student-specific measurement tool considered. Second, perceptions of FI as a normal part of the college experience may cause some students measured as food insecure to inaccurately perceive themselves as food secure. There is a need for validated measures of FI within the college setting due to several reported discrepancies of FI. Future work should attempt to develop such measures to better capture college student FI and provide the evidence needed to develop tailored interventions. 

## Figures and Tables

**Table 1:** Sociodemographic Characteristics of College Student Participants by AFSSM-Measured Food Security Status.

Variable	Total ^a^ n (%)	AFSSM-Measured Food Secure Mean (SD) or n (%)	AFSSM-Measured Food Insecure Mean (SD) or n (%)
**Age**	962	22.7 (5.0)	21.4 (3.7) ^+^
**Gender**	993	596 (60.0)	397 (40.0)
Male	335 (33.7)	210 (35.2)	125 (31.5)
Female	658 (66.3)	386 (64.8)	272 (68.5)
**Ethnicity**	996	599 (60.1)	397 (39.9)
Hispanic or Latino	225 (22.6)	111 (18.5)	114 (28.7) *
Not Hispanic or Latino	771 (77.4)	488 (81.5)	283 (71.3) *
**Race**	984	592 (60.2)	392 (39.8)
White	667 (67.8)	416 (70.3)	251 (64.0) *
Black or African American	71 (7.2)	21 (3.5)	50 (12.8) *
Asian	166 (16.9)	109 (18.4)	57 (14.5)
Multiple Races	58 (5.9)	36 (6.1)	22 (5.6)
Other Races	22 (2.2)	10 (1.7)	12 (3.1)
**Marital Status**	992	597 (60.2)	395 (39.8)
Single	827 (83.4)	490 (82.1)	337 (85.3)
Married	61 (6.1)	43 (7.2)	18 (4.6)
Living with Partner/In a Relationship	104 (10.5)	64 (10.7)	40 (10.1)
**Year in College**	1000	600 (60.0)	400 (40.0)
First Year	25 (2.5)	15 (2.5)	10 (2.5)
Second Year	595 (59.5)	362 (60.3)	233 (58.3)
Third Year	131 (13.1)	78 (13.0)	53 (13.3)
Four Year	113 (11.3)	46 (7.7)	67 (16.8) *
Graduate Student	136 (13.6)	99 (16.5)	37 (9.3) *
**Residence Status**	1002	601 (60.0)	401 (40.0)
In-state	824 (82.2)	490 (81.5)	334 (83.3)
Out-of-state	84 (8.4)	49 (8.2)	35 (8.7)
International	94 (9.4)	62 (10.3)	32 (8.0)
**Employment Status**	1001	600 (49.9)	401 (40.1)
Full-time (30 or more hours/week)	128 (12.8)	83 (13.8)	45 (11.2)
Part-time (1–29 h/week)	457 (45.7)	270 (45.0)	187 (46.6)
Not Employed	416 (41.6)	247 (41.2)	169 (42.1)
**Pell Grant Recipient**	995	600 (60.3)	395 (39.7)
Yes	284 (28.5)	120 (20.0)	164 (41.5) *
No	711 (71.5)	480 (80.0)	231 (58.5) *
**Place of Residence**	986	594 (60.2)	392 (39.8)
On-campus Residence Hall	141 (14.3)	89 (15.0)	52 (13.3)
Off-campus Housing	845 (85.7)	505 (85.0)	340 (86.7)
**Financial Independence**	1001	601 (60.0)	400 (40.0)
Yes	414 (41.4)	229 (38.1)	185 (46.3) *
No	587 (58.6)	372 (61.9)	215 (53.8) *

**^a^** Counts will not always sum to 1003 because of missing data. ^+^ Mean scores are significantly different between food secure and food insecure participants at *p*-value of <0.001 with independent samples *t*-test. * Proportion of participants is significantly different between food secure and food insecure groups at *p*-value < 0.05 with χ^2^ tests.

**Table 2:** Differences in USDA AFSSM Raw Scores by Self-Categorized Food Security Status among College Students AFSSM-Measured as Food Insecure (n = 401).

Variable	Total n (%)	AFSSM-Measured FI + Self-Categorized FS, n (%)	AFSSM-Measured FI + Self-Categorized FI, n (%)	*p*-Value *
**USDA AFSSM Raw Score Food Insecure Categories**	n = 401	n = 232	n = 169	<0.001
Raw Score of 3–5 (Low FS Category)	172 (42.9)	124 (53.4)	48 (28.4)	
Raw Score of 6–10 (Very Low FS Category)	229 (57.1)	108 (46.6)	121 (71.6)	

* Significantly different at *p*-value < 0.05 with χ^2^ test (χ^2^ (1, n = 401) = 25.0, *p* < 0.001). AFSSM-Measured FI + Self-Categorized FS = Measured as food insecure and categorized themselves as food secure (MFI-SFS); AFSSM-Measured FI + Self-Categorized FI = Measured as food insecure and categorized themselves as food insecure (MFI-SFI).

**Table 3:** Differences in Coping Strategies Subscale Scores by Self-Categorized Food Security Status among College Students AFSSM-Measured as Food Insecure.

Variable	Total ^a^, n	AFSSM-Measured FI + Self-Categorized FS n, Mean (SD)	AFSSM-Measured FI + Self-Categorized FI n, Mean (SD)	*p*-Value *
**Coping Strategies Mean Scores**				
Saving Subscale (max score of 27)	396	229, 17.41 (3.74)	167, 18.81 (3.52)	<0.001
Support Subscale (max score of 30)	400	231, 16.09 (2.92)	169, 16.56 (3.28)	0.13
Access Subscale (max score of 15)	397	229, 8.38 (1.72)	168, 9.08 (1.57)	<0.001
Selling Subscale (max score of 12)	395	227, 4.96 (1.40)	168, 5.35 (1.58)	0.01
Overall Score (max score of 84)	388	223, 46.92 (7.50)	165, 49.76 (7.54)	<0.001

* Significantly different at *p*-value < 0.05 with independent samples *t*-test; AFSSM-Measured FI + Self-Categorized FS = Measured as food insecure and categorized themselves as food secure (MFI-SFS); AFSSM-Measured FI + Self-Categorized FI = Measured as food insecure and categorized themselves as food insecure (MFI-SFI). ^a^ Counts will not always sum to 401 because of missing data.

**Table 4:** Differences in Overall Coping Strategies Scale Score by Self-Categorized Food Security Status among College Students AFSSM-Measured as Food Insecure (n = 388).

Variable	Total ^a^ n (%)	AFSSM-Measured FI + Self-Categorized FS n (%)	AFSSM-Measured FI + Self-Categorized FI n (%)	*p*-Value *
**Coping Strategies Overall Score by Quartiles**	n = 388	n = 223	n = 165	0.002
Score of 28.0–42.0 (bottom 25%)	101 (26.0)	70 (31.4)	31 (18.8)	
Score of 42.1–47.0	96 (24.7)	58 (26.0)	38 (23.0)	
Score of 47.1–53.0	107 (27.6)	60 (26.9)	47 (28.5)	
Score of 53.1–84.0 (top 25%)	84 (21.6)	35 (15.7)	49 (29.7)	

* Significantly different at *p*-value < 0.05 with χ^2^ test (χ^2^ (3, n = 388) = 14.8, *p* = 0.002). AFSSM-Measured FI + Self-Categorized FS = Measured as food insecure and categorized themselves as food secure (MFI-SFS); AFSSM-Measured FI + Self-Categorized FI = Measured as food insecure and categorized themselves as food insecure (MFI-SFI). ^a^ Counts do not sum to 401 because of missing data.

## Data Availability

The data presented in this study are available on request from the corresponding author. The data are not publicly available due to FERPA regulations.

## Supplementary Materials

The following supporting information can be downloaded at: https://www.mdpi.com/article/10.3390/nu14173569/s1, Table S1: Differences in Question Responses to USDA AFSSM by Self-Categorized Food Security Status among Students AFSSM-Measured as Food Insecure; Table S2: Differences in Total Coping Strategies Scale Scores by Self-Categorized Food Security Status among Students AFSSM-Measured as Having Low and Very Low Food Security; Table S3: Differences in Coping Strategies by Self-Categorized Food Security Status among Students AFSSM-Measured as Food Insecure.
