# Supplementary material for: Differences in Measured and Self-Categorized Food Security Status and Related Coping Strategies among College Students
Source: Nutrients. 2022 Aug 30;14(17):3569. doi: 10.3390/nu14173569 (PMC9460733; doi:10.3390/nu14173569)
Supplement: Supplementary file 1 [file nutrients-14-03569-s001.zip › Nutrients Table S1 Supplemental.pdf]

Supplemental Table S1. Differences in Question Responses to USDA AFSSM by Self-Categorized Food Security Status among Students AFSSM-Measured as Food Insecure

Variable	Total<br>n (%)<br>n=401	AFSSM-Measured<br>FI + Self-Categorized FS<br>n = 232 (57.9%)	AFSSM-Measured<br>FI + Self-Categorized FI<br>n = 169 (42.1%)	p-value*
**Question HH1: Which of these statements best describes the food eaten in your household in the last 12 months:**	n=401			**< 0.001**
Enough of the kinds of food we want to eat	99 (24.7)	80 (34.5)	19 (11.2)	
Enough but not always the kinds of food we want	232 (57.9)	130 (56.0)	102 (60.4)	
Sometimes not enough to eat	62 (15.5)	21 (9.1)	41 (24.3)	
Often not enough to eat	8 (2.0)	1 (0.4)	7 (4.1)	
**Question HH2: "I worried whether my food would run out before I got money to buy more."**	n=400			**< 0.001**
Often true	85 (21.3)	30 (13.0)	55 (32.5)	
Sometimes true	254 (63.5)	157 (68.0)	97 (57.4)	
Never true	61 (15.3)	44 (19.0)	17 (10.1)	
**Question HH3: "The food that I bought just didn't last, and I didn't have money to get more."**	n=397			**< 0.001**
Often true	54 (13.6)	19 (8.3)	35 (20.8)	
Sometimes true	225 (56.7)	125 (54.6)	100 (59.5)	
Never true	118 (29.7)	85 (37.1)	33 (19.7)	
**Question HH4: "I couldn't afford to eat balanced meals."**	n=401			**< 0.001**
Often true	162 (40.4)	67 (28.9)	95 (56.2)	
Sometimes true	201 (50.1)	136 (58.6)	65 (38.5)	
Never true	38 (9.5)	29 (12.5)	9 (5.3)	
**Question AD1: In the last 12 months, did you ever cut the size of your meals or skip meals because there wasn't enough money for food?**	n=400			**0.009**
Yes	343 (85.8)	189 (81.8)	154 (91.1)	

No	57 (14.2)	42 (18.2)	15 (8.9)	
**Question AD1a: If yes above, how often did this happen?**	n=343			**< 0.001**
Almost every month	97 (28.3)	33 (17.5)	64 (41.5)	
Some months but not every month	149 (43.4)	89 (47.1)	60 (39.0)	
Only 1 or 2 months	97 (28.3)	67 (35.4)	30 (19.5)	
**Question AD2: In the last 12 months, did you ever eat less than you felt you should because there wasn't enough money for food?**	n=400			**< 0.001**
Yes	320 (80.0)	171 (74.0)	149 (88.2)	
No	80 (20.0)	60 (26.0)	20 (11.8)	
**Question AD3: In the last 12 months, were you ever hungry but didn't eat because there wasn't enough money for food?**	n=397			**0.02**
Yes	261 (65.7)	141 (61.0)	120 (72.3)	
No	136 (34.3)	90 (39.0)	46 (27.7)	
**Question AD4: In the last 12 months, did you lose weight because there wasn't enough money for food?**	n=399			0.20
Yes	140 (35.1)	75 (32.5)	65 (38.7)	
No	259 (64.9)	156 (67.5)	103 (61.3)	
**Question AD5: In the last 12 months, did you ever not eat for a whole day because there wasn't enough money for food?**	n=381			**0.05**
Yes	59 (15.5)	27 (12.3)	32 (19.8)	
No	322 (84.5)	192 (87.7)	130 (80.2)	
**Question AD5a: If yes above, how often did this happen?**	n=58			0.10
Almost every month	13 (22.4)	3 (11.1)	10 (32.3)	
Some months but not every month	29 (50.0)	14 (51.9)	15 (48.4)	
Only 1 or 2 months	16 (27.6)	10 (37.0)	6 (19.3)	

*Significantly different at p-value <0.05 with χ^2 test. HH1: χ^2 (3, n=401)=43.1, p<0.001; HH2: χ^2 (2, n=400)=24.5, p<0.001; HH3: χ^2 (2, n=397)=21.6, p<0.001; HH4: χ^2 (2, n=401)=31.3, p<0.001; AD1: χ^2 (1, n=400)=6.9, p=0.009; AD1a: χ^2 (2, n=343)=26.4, p<0.001; AD2: χ^2 (1, n=400)=12.2, p<0.001; AD3: χ^2 (1, n=397)=5.4, p=0.02; AD5: χ^2 (1, n=381)=3.9, p=0.05.
