# Supplementary material for: Differences in Measured and Self-Categorized Food Security Status and Related Coping Strategies among College Students
Source: Nutrients. 2022 Aug 30;14(17):3569. doi: 10.3390/nu14173569 (PMC9460733; doi:10.3390/nu14173569)
Supplement: Supplementary file 1 [file nutrients-14-03569-s001.zip › Nutrients Table S2 Supplemental.pdf]

Supplemental Table S2. Differences in Total Coping Strategies Scale Scores by Self-Categorized Food Security Status among Students AFSSM-Measured as Having Low and Very Low Food Security

**Variable**	**Total n (%)**	**AFSSM-Measured Low FS + Self-Categorized FS**	**AFSSM-Measured Low FS + Self-Categorized FI**	**AFSSM-Measured Very Low FS + Self-Categorized FS**	**AFSSM-Measured Very Low FS + Self-Categorized FI**	**p-value***
**Coping Strategies Overall Score by Quartiles**	n=388	n=117	n=46	n=106	n=119	**<0.001**
Score of 28.0-42.0 (bottom 25%)	101 (26.0)	43 (36.8) [a]	12 (26.1) [a]	27 (25.5) [a]	19 (16.0) [b]	
Score of 42.1-47.0	96 (24.7)	38 (32.5)	15 (32.6)	20 (18.9)	23 (19.3)	
Score of 47.1-53.0	107 (27.7)	25 (21.4)	12 (26.1)	35 (33.0)	35 (29.4)	
Score of 53.1-84.0 (top 25%)	84 (21.6)	11 (9.3) [a]	7 (15.2) [a,]	24 (22.6) [a]	42 (35.3) [b]	

*Significantly different at p-value <0.05 with χ^2 test (χ^2 (9, n=388)= 38.9, p<0.001). Each subscript letter denotes a subset of Food Security Status categories whose column proportions do not differ significantly from each other at p=0.002; Measured Low FS + Self-Identified FS = Measured as having low food security and self-identified as food secure (low FS-SFS); Measured Low FS + Self-Identified FI = Measured as having low food security and self-identified as food insecure (low FS-SFI); Measured Very Low FS + Self-Identified FS = Measured as having very low food security and self-identified as food secure (very low FS-SFS); Measured Very Low FS + Self-Identified FI = Measured as having very low food security and self-identified as food insecure (very low FS-SFI). Bonferroni correction for post-hoc analysis: 0.05/24 = 0.002
