# Supplementary material for: Differences in Measured and Self-Categorized Food Security Status and Related Coping Strategies among College Students
Source: Nutrients. 2022 Aug 30;14(17):3569. doi: 10.3390/nu14173569 (PMC9460733; doi:10.3390/nu14173569)
Supplement: Supplementary file 1 [file nutrients-14-03569-s001.zip › Nutrients Table S3 Supplemental.pdf]

Supplemental Table S3. Differences in Coping Strategies by Self-Categorized Food Security Status among Students AFSSM-Measured as Food Insecure

Variable	Total n (%) n=401	AFSSM-Measured FI + Self-Categorized FS n = 232 (57.9%)	AFSSM-Measured FI + Self-Categorized FI n = 169 (42.1%)	p-value*
**Coping Strategies Saving Subscale**				
**Took fewer classes**	n=399			**N/A**
Often	10 (2.5)	4 (1.7)	6 (3.6)	
Sometimes	35 (8.8)	13 (5.6)	22 (13.2)	
Never	354 (88.7)	215 (92.7)	139 (83.2)	
**Used less utilities (electricity, water)**	n=401			**0.004**
Often	124 (30.9)	58 (25.0)	66 (39.1)	
Sometimes	111 (27.7)	64 (27.6)	47 (27.8)	
Never	166 (41.4)	110 (47.4)	56 (33.1)	
**Shared rent with others**	n=400			0.407
Often	167 (41.8)	90 (39.0)	77 (45.5)	
Sometimes	74 (18.5)	44 (19.0)	30 (17.8)	
Never	159 (39.8)	97 (42.0)	62 (36.7)	
**Planned menus before buying food**	n=400			0.360
Often	254 (63.5)	140 (60.6)	114 (67.4)	
Sometimes	108 (27.0)	68 (29.4)	40 (23.7)	
Never	38 (9.5)	23 (10.0)	15 (8.9)	
**Cut out food coupons**	n=400			0.082
Often	176 (44.0)	91 (39.4)	85 (50.3)	
Sometimes	155 (38.8)	95 (41.1)	60 (35.5)	
Never	69 (17.2)	45 (19.5)	24 (14.2)	
**Saved money on meds or medical appointments**	n=400			**< 0.001**
Often	110 (27.5)	55 (23.8)	55 (32.5)	
Sometimes	91 (22.8)	41 (17.8)	50 (29.6)	
Never	199 (49.8)	135 (58.4)	64 (37.9)	

**Stretched food to last longer**	n=401			**0.017**
Often	224 (55.9)	117 (50.5)	107 (63.3)	
Sometimes	146 (36.4)	98 (42.2)	48 (28.4)	
Never	31 (7.7)	17 (7.3)	14 (8.3)	
**Shared groceries/meals with roommates**	n=401			0.583
Often	144 (35.9)	86 (37.1)	58 (34.3)	
Sometimes	133 (33.2)	79 (34.0)	54 (32.0)	
Never	124 (30.9)	67 (28.9)	57 (33.7)	
**Save food for emergencies**	n=401			**< 0.001**
Often	92 (22.9)	38 (16.4)	54 (32.0)	
Sometimes	153 (38.2)	89 (38.3)	64 (37.9)	
Never	156 (38.9)	105 (45.3)	51 (30.1)	
**Coping Strategies Support Subscale**				
**Participated in paid research study to buy food**	n=401			0.088
Often	40 (10.0)	17 (7.3)	23 (13.6)	
Sometimes	84 (20.9)	53 (22.8)	31 (18.4)	
Never	277 (69.1)	162 (69.9)	115 (68.0)	
**Borrowed money from family/friends**	n=401			0.242
Often	62 (15.5)	30 (12.9)	32 (19.0)	
Sometimes	175 (43.6)	106 (45.7)	69 (40.8)	
Never	164 (40.9)	96 (41.4)	68 (40.2)	
**Attended functions where there was free food**	n=401			0.365
Often	187 (46.6)	102 (44.0)	85 (50.3)	
Sometimes	187 (46.6)	112 (48.3)	75 (44.4)	
Never	27 (6.8)	18 (7.7)	9 (5.3)	
**Obtained food from food bank or pantry**	n=401			**0.049**
Often	37 (9.2)	23 (9.9)	14 (8.3)	
Sometimes	77 (19.2)	35 (15.1)	42 (24.9)	
Never	287 (71.6)	174 (75.0)	113 (66.8)	
**Participated in food assistance program (e.g. SNAP; WIC)**	n=401			0.912
Often	12 (3.0)	7 (3.0)	5 (3.0)	

Sometimes	42 (10.5)	23 (9.9)	19 (11.2)	
Never	347 (86.5)	202 (87.1)	145 (85.8)	
**Ate meals at places where you pay what you can**	n=401			0.955
Often	31 (7.7)	18 (7.8)	13 (7.7)	
Sometimes	57 (14.2)	34 (14.6)	23 (13.6)	
Never	313 (78.1)	180 (77.6)	133 (78.7)	
**Joined a group where free meals are provided**	n=401			0.868
Often	24 (6.0)	13 (5.6)	11 (6.5)	
Sometimes	75 (18.7)	45 (19.4)	30 (17.8)	
Never	302 (75.3)	174 (75.0)	128 (75.7)	
**Visited family on weekends to bring food back to school**	n=401			0.749
Often	79 (19.7)	44 (19.0)	35 (20.7)	
Sometimes	146 (36.4)	88 (37.9)	58 (34.3)	
Never	176 (43.9)	100 (43.1)	76 (45.0)	
**Held ≥1 part- or full-time jobs**	n=401			0.101
Often	124 (30.9)	71 (30.6)	53 (31.4)	
Sometimes	97 (24.2)	48 (20.7)	49 (29.0)	
Never	180 (44.9)	113 (48.7)	67 (39.6)	
**Used credit card to buy food**	n=400			0.317
Often	148 (37.0)	79 (34.2)	69 (40.8)	
Sometimes	87 (21.7)	55 (23.8)	32 (18.9)	
Never	165 (41.3)	97 (42.0)	68 (40.3)	
**Coping Strategies Access Subscale**				
**Ate less healthy meals to eat more food**	n=401			**< 0.001**
Often	207 (51.6)	92 (39.7)	115 (68.1)	
Sometimes	150 (37.4)	105 (45.2)	45 (26.6)	
Never	44 (11.0)	35 (15.1)	9 (5.3)	
**Took food home from on-campus dining hall**	n=399			0.208
Often	71 (17.8)	46 (20.0)	25 (14.8)	
Sometimes	102 (25.6)	62 (27.0)	40 (23.7)	
Never	226 (56.5)	122 (53.0)	104 (61.5)	

**Obtained food from dumpster or trash**	n=398			N/A
Often	2 (0.5)	1 (0.4)	1 (0.6)	
Sometimes	11 (2.8)	7 (3.1)	4 (2.4)	
Never	385 (96.7)	221 (96.5)	164 (97.0)	
**Purchased cheap, processed food**	n=398			**< 0.001**
Often	201 (50.5)	88 (38.3)	113 (67.3)	
Sometimes	161 (40.5)	114 (49.6)	47 (28.0)	
Never	36 (9.0)	28 (12.1)	8 (4.7)	
**Bartered services/items to buy food**	n=400			0.271
Often	17 (4.3)	7 (3.0)	10 (5.9)	
Sometimes	47 (11.7)	25 (10.8)	22 (13.0)	
Never	336 (84.0)	199 (86.2)	137 (81.1)	
**Coping Strategies Selling Subscale**				
**Sold textbooks**	n=398			0.560
Often	45 (11.3)	24 (10.5)	21 (12.4)	
Sometimes	80 (20.1)	43 (18.8)	37 (21.9)	
Never	273 (68.6)	162 (70.7)	111 (65.7)	
**Sold personal possessions**	n=400			**0.005**
Often	41 (10.3)	16 (6.9)	25 (14.8)	
Sometimes	110 (27.5)	57 (24.7)	53 (31.4)	
Never	249 (62.3)	158 (68.4)	91 (53.8)	
**Sold blood/plasma to buy food**	n=399			0.094
Often	21 (5.3)	9 (3.9)	12 (7.2)	
Sometimes	35 (8.7)	16 (6.9)	19 (11.3)	
Never	343 (86.0)	206 (89.2)	137 (81.5)	
**Sold sperm/eggs to buy food**	n=398			N/A
Often	1 (0.3)	1 (0.4)	0	
Sometimes	6 (1.5)	5 (2.2)	1 (0.6)	
Never	391 (98.2)	223 (97.4)	168 (99.4)	

*Significantly different at p-value <0.05 with χ^2 test.
